# Photodynamic therapy and associated targeting methods for treatment of brain cancer

**DOI:** 10.3389/fphar.2023.1250699

**Published:** 2023-09-28

**Authors:** Dorota Bartusik-Aebisher, Iga Serafin, Klaudia Dynarowicz, David Aebisher

**Affiliations:** ^1^ Department of Biochemistry and General Chemistry, Medical College of the University of Rzeszów, Rzeszów, Poland; ^2^ Students English Division Science Club, Medical College of the University of Rzeszów, Rzeszów, Poland; ^3^ Center for Innovative Research in Medical and Natural Sciences, Medical College of the University of Rzeszów, Rzeszów, Poland; ^4^ Department of Photomedicine and Physical Chemistry, Medical College of the University of Rzeszów, Rzeszów, Poland

**Keywords:** PDT, brain cancer, glioblastoma, diagnostics, treatment

## Abstract

Brain tumors, including glioblastoma multiforme, are currently a cause of suffering and death of tens of thousands of people worldwide. Despite advances in clinical treatment, the average patient survival time from the moment of diagnosis of glioblastoma multiforme and application of standard treatment methods such as surgical resection, radio- and chemotherapy, is less than 4 years. The continuing development of new therapeutic methods for targeting and treating brain tumors may extend life and provide greater comfort to patients. One such developing therapeutic method is photodynamic therapy. Photodynamic therapy is a progressive method of therapy used in dermatology, dentistry, ophthalmology, and has found use as an antimicrobial agent. It has also found wide application in photodiagnosis. Photodynamic therapy requires the presence of three necessary components: a clinically approved photosensitizer, oxygen and light. This paper is a review of selected literature from Pubmed and Scopus scientific databases in the field of photodynamic therapy in brain tumors with an emphasis on glioblastoma treatment.

## 1 Introduction

Malignant tumors of the brain and central nervous system (CNS) are a serious threat to health and cause the death of tens of thousands of people every year around the world (Ostrom et al., 2021). According to Ostrom et al., 83,029 deaths due to malignant brain tumors and other CNS tumors were recorded in 2014–2018 alone (Ostrom et al., 2021). In 2020, according to World Health Organization (WHO) reports, there were 308,102 new cases and 251,329 deaths due to malignant brain tumors, of which more than 50% of cases occur in Asia. In Poland in 2020, 4,413 new diagnoses were made (Raporty WHO, 2023). According to Fan et al., in 2019 alone, 347,992 cases of brain and CNS tumors were registered worldwide. This represents an increase of 94.35% compared to the period between the years 1990–2019 (Fan et al., 2022).

The most frequently diagnosed malignancy within the CNS is glioblastoma multiforme (GBM) which accounts for about 70% of all intracranial tumors with malignancy grade IV according to WHO classifications. It is estimated that in 80% of cases, tumor recurrence occurs after tumor resection, which makes the prognosis poor, despite the development of surgical techniques, as well as adjuvant radio- and chemotherapy, which are standard therapeutic procedures. The most common surgical procedures include: biopsy (i.e., taking a piece of tissue and analyzing it under a microscope), neurosurgical resection, partial or complete resection. The most commonly used radiotherapy is stereotactic radiotherapy, i.e., external radiotherapy. Depending on the type of dose application, we distinguish stereotactic radiosurgery (single dose) or fractionated stereotactic radiotherapy (several doses) (Fuentes et al., 2018). The application of a specific method depends to a large extent on the location of the tumor, the size and extent of the lesions. Other radiotherapy techniques for the treatment of brain tumors are: intensity modulated radiotherapy technique, volumetric modulated arc therapy. All these techniques enable the application of higher radioactive doses compared to conventional radiotherapy (Scaringi et al., 2018). Proton therapy, on the other hand, uses proton radiation to destroy dangerous lesions. In turn, in chemotherapy, the most commonly administered drugs are: tucatinib, adotrastuzumab emtansine, trastuzumab deruxtekan and neratinib, which show intracranial efficacy. The development of new therapeutic drugs and innovative strategies are still being developed (Newton, 2000; Quirk et al., 2015; Bartusik-Aebisher et al., 2022; Corti et al., 2022). Due to the infiltration of GBM into adjacent tissues, complete resection is difficult or even impossible due to the need to remove an appropriate margin of brain tissue, which may result in serious neurological consequences for the patient. On the other hand, traditional treatment involving irradiation and the use of chemotherapeutics carries many side effects, including the risk of damage to healthy tissues resulting from their low precision (Mahmoudi et al., 2019). For these reasons, the method of photodynamic therapy (PDT) is increasingly considered as an alternative.

**FIGURE 1:**
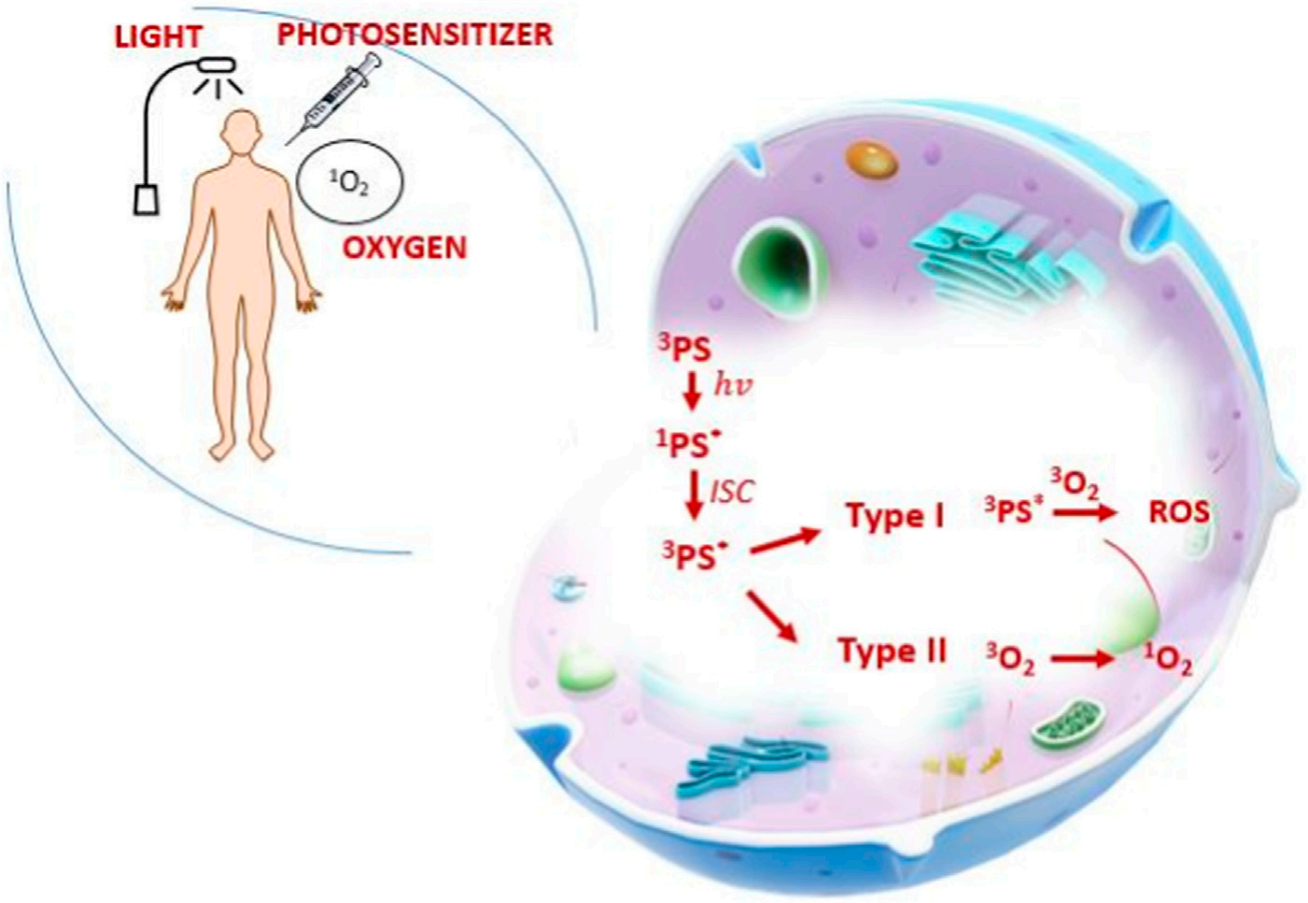
Mechanism of PDT.

The main principle of operation of PDT is based on the effect of combining photosensitizer molecules with cancer cells and their activation by excitation with laser light of the appropriate wavelength. The excited photosensitizer converts molecular oxygen to the singlet state as a result of the intersystem transition. This active form of oxygen generates a large amount of reactive oxygen species (ROS) (Bartusik-Aebisher et al., 2022). The generated reactive forms cause the oxidation of biological macromolecules (including proteins, fatty acids and DNA), consequently leading to damage to cellular organelles, i.e., lysosomes, nucleus, mitochondria, leading the cell to the path of programmed cell death (Xu et al., 2022). Figure 1 shows mechanism of PDT.

Photodynamic therapy is a progressive method of therapy used in dermatology, dentistry, ophthalmology and brain cancer**.** In dermatology, PDT is used in various types of skin diseases. Examples include: actinic keratosis (Mazur and Reich, 2023), acne vulgaris (Wang et al., 2023), port-wine stains (Kang et al., 2023) or more or less advanced skin cancers. In the treatment of dermatological diseases, the most often uses a cream with a component of one of the photosensitizers. On the other hand, in the treatment of brain tumors, the photosensitizer is administered systemically or orally (Hsia et al., 2023)**.** The type of light source is adapted to the applied photosensitizer. Both in dermatological cases and brain tumors, the wavelength is in the range of 500–680 nm. The form of laser light delivery in dermatology takes place locally and centrally in the lesion area. However, in the case of brain tumors, a fiber optic diffuser, optical fiber, cavitation balloon, i.e., interstitial application, are used. Therapeutic effectiveness in the treatment of brain tumors depends on the forms of light application as well as on the geometry of the light. LED diodes are used more and more often, which prevent the tissue from heating up under the influence of the laser. In dermatological cases, the irradiation time varies from a few minutes to several minutes. According to Li et al., who conducted a systematic review of PDT in the treatment of rosacea, most studies showed satisfactory treatment effects (Li et al., 2022). In the case of brain tumor therapy, the effectiveness varies. According to Jamali et al., the number of killed human glioblastoma cells was higher after exposure to the blue LED compared to the red one (Jamali et al., 2018). Many studies and experiments have been developed to analyze the effectiveness of PDT in dentistry. PDT in dentistry is used in such diseases as: halitosis, biofilm, caries (Mazur et al., 2022). The average effectiveness of PDT in the treatment of oral lesions is 90%. Methylene Blue is a frequently used photosensitizer in dentistry and the light sources used have a wavelength in the range of 600–630 nm. The laser is applied directly to the changes or inserted into the periodontal pocket (Gholami et al., 2023) In turn, in ophthalmology, PDT is used to treat diseases such as: keratitis (de Paiva et al., 2022), treatment of choroidal melanoma (Kawczyk-Krupka et al., 2013) or hemangioma (Kumar et al., 2022). In ophthalmology, the most commonly used photosensitizers are: riboflavin, methylene blue and verteporfin (de Paiva et al., 2022). The most commonly used light source is ultraviolet and light-emitting diodes. There are numerous studies confirming the effectiveness of PDT in eye diseases. In the case of choroidal melanoma (studies led by Soucek and Cihelkova, PDT resulted in complete tumor regression (Soucek and Cihelkova, 2006). According to Kawczyk-krupka et al., Clinical trials on the application of PDT in ophthalmology should still be conducted (Kawczyk-Krupka et al., 2013).

In PDT photosensitizer (PS) accumulates in cancer cells prior to irradiation causing the production of toxic reactive oxygen species (ROS) that destroy abnormal tissue. Tumor destruction occurs in three primary ways: direct killing of the tumor cell, damage to the tumor vasculature, and activation of the body’s immune response (Dolmans et al., 2003). The effect of photodynamic therapy is affected by the modification of any of the PDT components (photosensitizer, visible light, reactive oxygen species), phenotypic variability of cancer cells and variability of the tumor environment. Different combinations of photosensitizers with appropriate beams of light produce different results. An ideal photosensitizer should absorb light in the red or far red wavelength, and must also be able to penetrate the blood-brain barrier. It should be highly selective towards tumor tissue, low-toxic and quickly eliminated from the body (Tetard et al., 2014). The effectiveness of the therapy is directly proportional to the amount of singlet oxygen produced after the use of the appropriate wavelength of light (Stylli and Andrew, 2006).

The aim of this study was to review the literature on targeted methods of treating brain tumors (primarily glioblastoma multiforme), with particular emphasis on the currently developed photodynamic therapy (taking into account the mechanism of its action, the impact of individual components) and immunotherapy.

## 2 Materials and methods

A search focused on the use photodynamic therapy and other targeted methods in the treatment of a brain tumor was conducted on Pubmed, and Scopus from 1990 to 2023. The search term included the phrase: “photodynamic therapy in brain cancer”, “targeted methods in the treatment of a brain tumor”. The authors of this review worked on the basis of an agreed scheme, selecting articles based on their title, language, abstract, and access. This review was conducted based on the Preferred Reporting Items for Systematic Reviews and Meta-Analyses (PRISMA) guidelines (Page et al., 2021). Full-text and accessible articles were reviewed.

**FIGURE 2:**
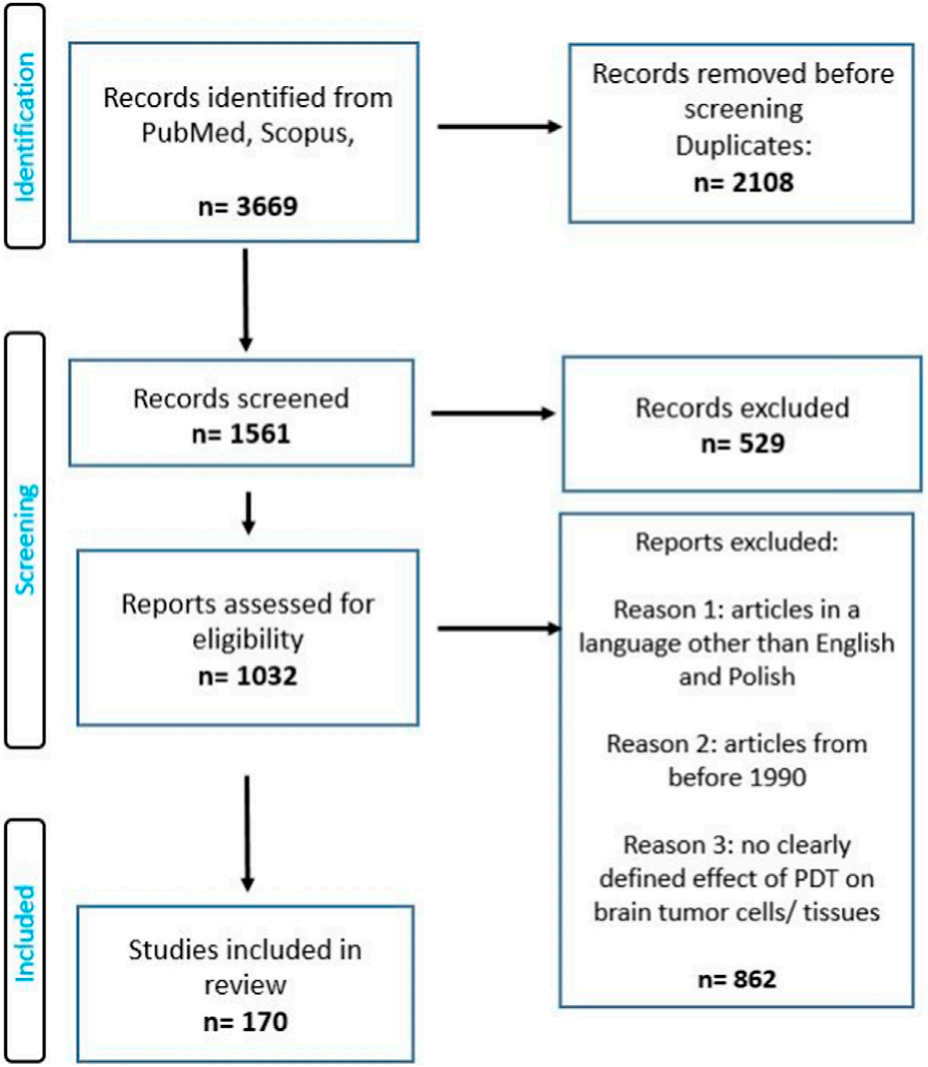
PRISMA flow diagram of included studies.

In the selection of articles, inclusion and exclusion criteria were used (Figure 2).

Inclusion.

• all types of brain cancer
• qualifying both *in vivo* and *in vitro* studies

Exclusion.

• articles in a language other than English or Polish
• articles from before 1990
• no clearly defined effect of PDT on brain tumor cells/tissues

## 3 Results and discussion

### 3.1 Malignant neoplasms of the CNS

**FIGURE 3:**
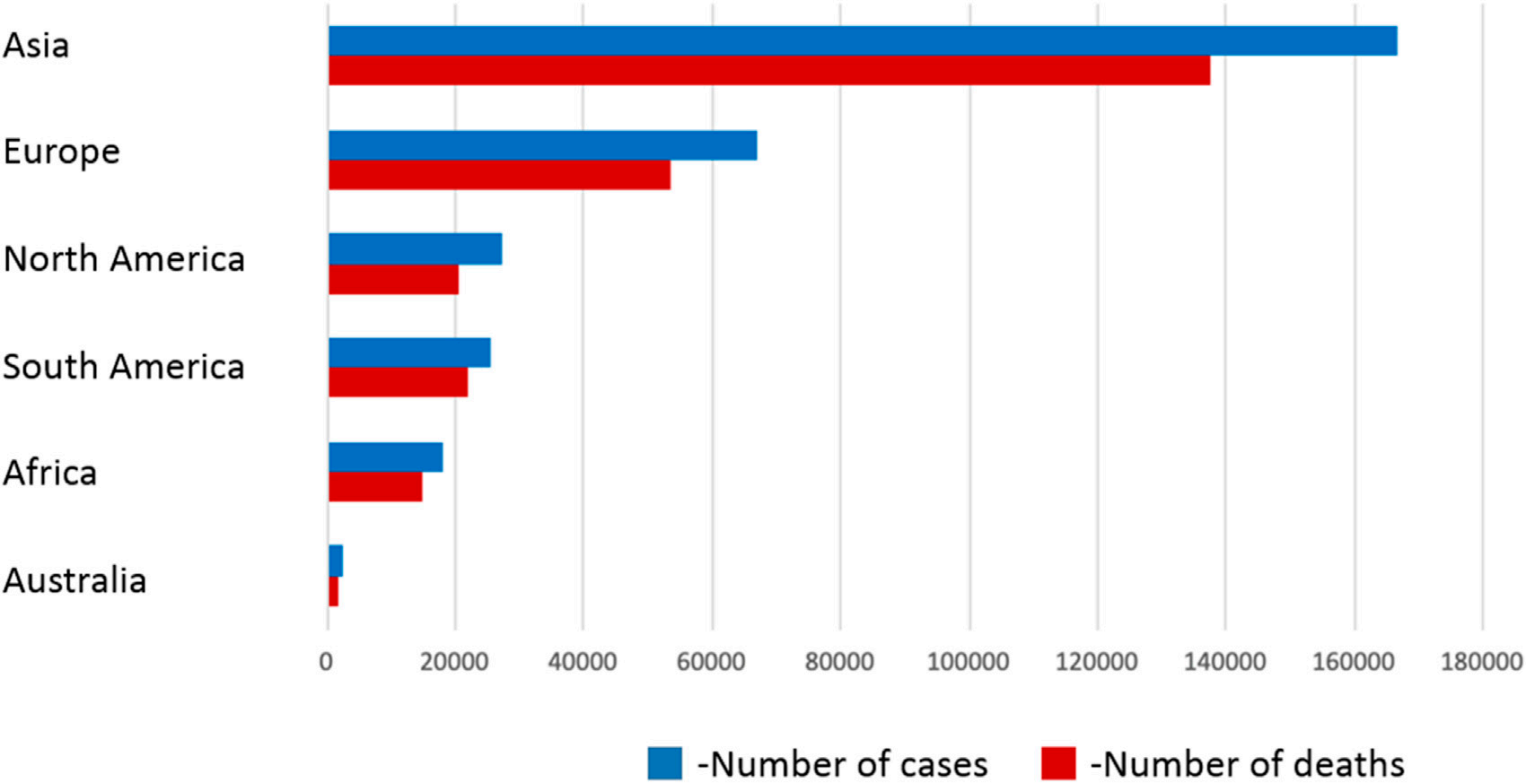
Estimated total number of cases and deaths due to brain and CNS tumors in 2020 in various regions of the world. Source: WHO Reports (Louis et al., 2021)

Central nervous system tumors include primary tumors (benign and malignant) and metastatic tumors located within the spinal cord and brain. Primary tumors of this system have characteristic features, such as no detectable pre-invasive or *in situ* features, no metastasis outside the CNS, possibility of dissemination through the cerebrospinal fluid (especially anaplastic tumors), and the location of the tumor has a key impact on prognosis. In children, tumors most often arise in the posterior cranial fossa, while in adults, the supratentorial. According to the WHO classification, CNS tumors can be divided into primary glioblastomas, peripheral nerve tumors, embryonic tumors, meningeal tumors, cardiovascular lymphomas, germline tumors, and metastatic tumors. Gliomas, classified on the basis of their similarity to various types of glial cells, include astrocytomas (including grade IV glioblastoma multiforme), oligodendrogliomas, and ependymomas. Poor prognosis is attributed to embryonic neoplasms, all of which are grade IV, especially medulloblastoma located in the cerebellum and occurring mainly in children. On the other hand, meningiomas are usually benign tumors and usually appear in adults (Fujisawa et al., 2000; Holland, 2000; Maher et al., 2006; Furnari et al., 2007; Kanu et al., 2009; Wirsching et al., 2016; Shah and Kochar, 2018; DeCordova et al., 2020; Ma et al., 2021; Martin et al., 2021). Despite medical progress, the average survival time of patients from the moment of diagnosis of GBM using standard treatment methods, such as surgical resection, radio- and chemotherapy, is less than 15 months (The median survival depends largely on the MGMT methylation state of the GBM and 15 months is the worst-case scenario) (Awad and Sloan, 2014), and in combination with temozolomide it ranges from 31.4 months to 48.1 months (DeCordova et al., 2020; Lazaridis et al., 2022). Factors contributing to poor prognosis include late diagnosis of advanced cancer, diffuse and invasive infiltration, pseudonecrosis, microvascular proliferation and resistance to conventional treatment, as well as heterogeneity of tumor cells and tumor microenvironment. Glioblastoma multiforme is usually located in the frontal or temporal lobes, rarely in the brainstem, cerebellum or spinal cord. In approximately 13% of cases, GBM occurs as multifocal or multicentric masses (more than two lesions, including the meninges), may form distant lesions or occur in a diffuse form (Khandwala et al., 2021). About 90% of cases of GBM are primary tumors, developing *de novo*, mainly in patients over 45 years of age. The remaining 10% develops within 5–10 years from a lower-grade malignancy (secondary GBM) and occurs mainly in patients under 45 years of age (Fujisawa et al., 2000; Holland, 2000; Maher et al., 2006; Furnari et al., 2007; Kanu et al., 2009; DeCordova et al., 2020). The symptoms of the disease depend on the area of the brain affected by the tumor. These include: headaches and dizziness, nausea, vomiting, impaired cognitive functions, confusion, speech disorders (the most common symptoms), convulsions, persistent weakness and fatigue, numbness, loss of vision, impaired executive functions, mood disorders, changes in behavior and even personality or memory disorders (Chaichana et al., 2009; Alexander and Cloughesy, 2017; Dajani et al., 2022). According to the WHO (Louis et al., 2021) classification updated in 2016, GBM is divided into the following molecular subtypes (division due to the presence or absence of mutations in the isocitrate dehydrogenase gene IDH): I- wild type (without mutations) - about 90% of cases, it is primary or *de novo* and common in patients >55 years of age; II- GBM with IDH mutation (approximately 10% of cases), includes secondary GBM in patients with a history of previous low-grade glioma and often occurs in younger patients; this type has a better prognosis than type I; III- GBM, not otherwise specified (NOS) - a diagnosis intended for tumors for which a full IDH analysis cannot be performed (Batash et al., 2017). Primary GBM markers include epidermal growth factor receptor (EGFR) amplification, PTEN gene mutations (phosphatase and tensin homolog deleted on chromosome 10) and telomerase promoter with reverse transcriptase activity (TERT). Secondary GBM show mutations in the isocitrate dehydrogenase 1 and 2 (IDH1/2) gene, the p53 protein and the ATRX gene (Parsons et al., 2008; Rohle et al., 2013; Schumacher et al., 2014; Bush et al., 2017). Figure 3 shows estimated total number of cases and deaths due to brain and CNS tumors in 2020 in various regions of the world.

### 3.2 The use of photodynamic therapy in the treatment of brain tumors (GBM) and factors affecting the effect of therapy

In PDT irreversible destruction of neoplastic tissue can occur in several ways (de Paula et al., 2017). The first is direct cell death via apoptosis, necrotic cell death or autophagy, with apoptosis being the predominantly preferred mechanism (DUBEY et al., 2019). Apoptosis is characterized by nuclear fragmentation, chromatin condensation and formation of apoptotic bodies, while necrotic cell death is characterized by destruction of organelles, disruption of the plasma membrane and induction of an immune response (DUBEY et al., 2019). The second mechanism is the antivascular effect. Activation of photosensitizers leads to the destruction of endothelial cells, which in turn leads to the formation of thrombogenic sites, thus initiating a cascade of reactions leading to vessel closure or damage, leading to the death of cancer cells by depriving them of oxygen and nutrients (Velazquez, 2007). The third mechanism is the activation of the immune response against cancer cells through acute inflammatory processes and the release of cytokines into the tumor, which results in the influx of macrophages and leukocytes that can contribute to tumor destruction and stimulate the immune system to recognize and eliminate cancer cells (de Paula et al., 2017). These mechanisms are interrelated, and the predominance of one pathway over others depends on the parameters used in therapy as well as the disease state and health of the patient (Mroz et al., 2004).

Singlet oxygen has a short diffusion distance and short lifetime, which means that its activity is limited only to local tumor tissues without adversely affecting healthy tissues. According to Semyachkina-Glushkovskaya et al. and Ibarra et al., GBM cells are much more sensitive to the PDT method compared to other cancer cells (Semyachkina-Glushkovskaya et al., 2022a; Ibarra et al., 2021). Nevertheless, the effectiveness of photosensitizers is still not satisfactory, mainly due to their limitations.

#### 3.2.1 Other factors affecting the effect of therapy

The effectiveness of photodynamic therapy is also influenced by the conditions of the tumor environment. The efficiency of production and use of free oxygen radicals is often reduced by deteriorating hypoxic conditions in the tumor. Oxygen concentration in brain tissue is normally 5%–15%, while in gliomas it may reach 0.1% in a necrotic tumor (Ihata et al., 2022).

The use of analgesics such as phenytoin (PHY) *in vitro* seems to affect the effective-ness of photodynamic therapy. Studies using this drug in 5-ALA therapy indicate a de-crease in PpIX synthesis. However, the effectiveness of PDT does not seem to change (Hefti et al., 2012).

In addition, photodynamic therapy of glioblastoma is also affected by ambient tem-perature. The mere use of a laser in PDT increases the temperature of the tissue, however, the simultaneous exposure of the *in vitro* culture to hyperthermia (HT) results in a synergistic effect of both therapies. The degree of synergism increases with increasing temperature (Hirschberg et al., 2004).

Proteins associated with oxidative stress (superoxide dismutase, catalase, NO syn-thase), as well as glutathione and related substances (glutathione peroxidase, glutathione S-transferase, omega-1 glutathione transferase, glutathione synthase) and hemoxygen-ase-1 (HO -1), apurin/apyrimidine endonuclease 1/redox factor-1 (APE1/Ref-1) and others affect the resistance of cells to PDT (Shinoda et al., 2021).

Müller et al., research from 2020 proved the different susceptibility of glioblastoma multiforme cells (U251MG line) to PDT depending on the expression of the transporter for protoporphyrin IX - ABCG2. High expression of this receptor reduces the accumulation of the photosensitizer, and then a higher dose of light is required for the appropriate effect of phototherapy. However, in order to restore the appropriate susceptibility of the cell population, an appropriate antagonist for ABCG2 can be used, e.g., non-toxic KO143, gefitinib or lapatinib (Sun et al., 2013; Zhao et al., 2013; Müller et al., 2020; Mansi et al., 2022).

Equally important is the expression of mRNA of the ferrochelatase (FECH) gene, which is an enzyme that catalyzes the conversion of protoporphyrin IX to heme. Silencing FECH results in a marked inhibition of growth and induction of apoptosis by PDT in glioblastoma cells (Teng et al., 2011).

In addition, prior administration of certain substances may result in increased cell death after photodynamic therapy. Such substances include, among others, calcitriol, arsenic trioxide, methadone, motexafin gadolinium (MGd), Shikonin (Madsen et al., 2009; Chen et al., 2014; Werner et al., 2022).

The main advantage of this method is its high precision—cancer tissue or tumor vasculature containing PS can be selectively irradiated sparing healthy tissue (Eljamel, 2008; Eljamel, 2010). Another issue related to PDT is the possibility of using 5-aminolevulinic acid (5-ALA) for intraoperative imaging during brain tumor resection. This compound, localized in cancer cells after administration, enzymatically forms protoporphyrin IV which fluoresces under exposure to blue light making it easier for surgeons to detect residual cancer tissue precisely for minimizing cancer recurrence (Eljamel, 2010). Delivery of PS and other therapeutic drugs to the CNS may be hindered by the need to overcome the blood-brain barrier (BBB) - a physical obstacle for the transport of substances between the blood and the cerebral spinal fluid (CSF), created by closely adhering capillary endothelial cells (Eljamel, 2008; Eljamel, 2010). It has been discovered that PDT can be used to open the BBB (for more effective delivery of drugs), and also to enhance lymphatic drainage and purify the CNS of unnecessary substances (Semyachkina-Glushkovskaya et al., 2022b). Currently, various methods of transporting medicinal substances to the tumor are being developed, of which the use of nanoparticles as carriers seems promising. They reach the tumor microenvironment (TME) by combining with its components (including immune cells) and are able to modify them, including stimulating the body’s immune-anti-cancer reactions. Nanoparticles accumulate in the tumor due to leaky vasculature and damaged lymphatic drainage, which significantly increases the concentration of drugs in the diseased tissue and reduces side effects (Casas, 2020; Yang et al., 2020). Currently, much research is focused on the development of targeted GBM immunotherapy, aimed at “reprogramming” the immune system to fight cancer. First of all, the use of immune checkpoint inhibitors (ICIs) and T lymphocytes with chimeric antigen receptors (CARs), which have been effective in the treatment of other cancers, is being considered (Yu and Quail, 2021). Other developed methods of targeted treatment of brain tumors include, among others, vaccine and gene therapy, which consists in introducing modified genes into tumor cells in order to destroy them (Eljamel, 2008).

### 3.3 Efficiency and advantage of PDT in the treatment of glioblastoma - examples from the literature

Photodynamic therapy was first approved in 1993 in Canada using the photosensitizer Photofrin for the treatment of bladder cancer (Dolmans et al., 2003). Since then, many papers have been published confirming the advantages of PDT in the treatment of gliomas and showing its advantage over standard surgical therapy. Examples are shown below.

Observations showed that patients who underwent surgery combined with photodynamic therapy showed a longer survival period than those who underwent surgery alone (Bartusik-Aebisher et al., 2022). A 2011 study reported 73 male patients with GBM. They received standard therapy (ST), ST + PDT or ST + PDT + IORT (intraoperative radiotherapy). The mean survival of patients treated with PDT was significantly longer than those treated with ST alone (62.9 weeks vs. 20.6 weeks). IORT alone did not make a significant difference in survival (Lyons et al., 2012). Another study showed that after using PTD with 5-ALA in a group of 10 patients with unresectable recurrent gliomas, the median survival from 6 to 8 months increased to about 15 (Beck et al., 2007). Similarly, another study (Stummer et al., 2006) showed that intraoperative PDT with 5-aminolevulinic acid allowed for more accurate glioblastoma resection and increased the number of patients with 6-month recurrence-free survival from 21.1% to 41%. Similar conclusions were included in the study (Eljamel et al., 2008), where it was indicated that intraoperative use of 5-ALA to facilitate GBM resection and repeated use of PTD allowed to extend the average survival in the study group from 24.6 to 52.8 weeks and the average progression-free survival tumor from 4.8 to 8.6 months. A study (Stummer et al., 2008) describes a case of a patient with GBM, previously treated with surgery, radiotherapy and chemotherapy, who developed a neoplastic lesion in the insula, resistant to secondary treatment. PDT with 5-ALA was applied and the lesion disappeared after 24 h. At the time of writing the paper by the researchers, the patient has still not relapsed after 56 months of therapy, which is an impressive result. In a study (Stylli et al., 2005), the effect of PDT with the use of HpD on the survival of patients with anaplastic astrocytoma (AA) and GBM after surgical removal of the tumor was tested. For newly diagnosed GBM, the median survival from diagnosis was 14.3 months, and after treatment with PDT, 25% of patients with this glioma survived for more than 36 months. In the case of recurrent GBM, the median survival from the time of surgery was 13.5 months, after PDT treatment, the survival rate of 41% of patients was more than 36 months. These examples and many others testify to the progress and increase in the effectiveness of GBM treatment through the use of photodynamic therapy. Lietke et al., in turn, performed interstitial PDT in combination with 5-ALA in patients with recurrent malignant glioma. Median age of patients 49.4 years. The eligibility criterion was the presence of glioblastoma recurrence. The maximum lesion size was not to exceed 3 cm. A diode laser (wavelength 635 nm) was used as the light source. The applied photosensitizer was 5-ALA at a dose of 20 mg/kg of body weight. The duration of the procedure was 60 min. The therapeutic effect was analyzed by magnetic resonance imaging with contrast. One of the main findings of the study was that interstitial PDT (iPDT) appears to be associated with beneficial treatment outcomes even in heavily treated malignant glioma relapses (Lietke et al., 2021).

### 3.4 Photosensitizers - 5-aminolevulinic acid, protoporphyrin IX, photofrin

5-aminolevulinic acid (ALA) is an organic chemical compound from the group of keto acids and amino acids, which is a derivative of levulinic acid. It is a precursor to the synthesis of porphyrins, including heme. In a several-stage process, ALA is converted to protoporphyrin IX (PpIX) - an endogenous photosensitizer accumulating in cancer cells (therefore, 5-ALA and its derivatives are considered prodrugs in PDT) (Mahmoudi et al., 2019). Protoporphyrin IV is a photoactive compound, which is used during tumor imaging during surgery - after exposure to blue-violet light, it is possible to observe the fluorescence of tumor cells in the red light wavelength range. Activation of PpIX by red light combined with oxygen produces singlet oxygen and causes cell death by apoptosis and necrosis. In this case, the use of red light for irradiation is more beneficial due to better penetration into tissues at a wavelength of 632 nm (Eljamel et al., 2008). Accumulation of protoporphyrin in tumor tissues results from, among others, due to the fact that their cells are characterized by lower expression of ferrocelatase - an enzyme converting PpIX into heme (Yang et al., 2015). In addition, it was found that by affecting the expression of enzymes involved in the synthesis of PpIX, such as porphobilinogen synthase (PBGS) and porphobilinogen deaminase (PBGD), tissue susceptibility to PDT can be regulated (Yang et al., 2015). The fluorescent and photosensitizing properties of 5-ALA/PpIX are particularly useful in the treatment of HGG (high-grade gliomas) by both FGS (Fluorescence-guided surgery) with blue light activation and PDT with red light activation (Mahmoudi et al., 2019). The first method (5-ALA in FGS for HGG) was approved by the European Union, while the FDA recognized 5-ALA as the first ever fluorescent agent for intraoperative visualization of brain tumors, enabling more effective resection (Mahmoudi et al., 2019). Another common photosensitizer is Porfimer sodium with the trade name Photofrin, which is a hematoporphyrin (HpD) derivative. The hematoporphyrin derivative (HpD; Photofrin^®^) was the first photosensitizer to be thoroughly investigated (Tetard et al., 2014). It accumulates in cancer cells and is activated by laser light with a wavelength of 630 nm (red light) and contact with tissue oxygen to produce highly reactive excited singlet oxygen, which oxidizes cancer cell components, e.g., mitochondrial enzymes, and leads to their destruction. In addition, this compound is used to enhance the fluorescence of PpIX in the intraoperative treatment of gliomas (Eljamel et al., 2008; Yang et al., 2015). The selectivity of Photofrin in settling in tumor cells may be due to the increased expression of LDL receptors on their surface, which bind lipoproteins circulating in the blood, which are carriers of photosensitizers, including sodium porfimer (Candide et al., 1986; Korbelik, 1992; Korbelik, 1993; Tsukagoshi, 1995; Misawa et al., 2005). This mechanism, as well as the participation of other plasma proteins in the distribution of photosensitizers, creates a field for discussion and further research. Currently, PS molecules are divided into three generations of compounds. The first generation includes naturally occurring porphyrins - including hematoporphyrins and HpD (Photofrin^®^, a mixture of dimers and oligomers of porphyrins, also known as sodium porfimer). The second generation includes chlorides (sodium talaporfin and temoporfin), benzoporphyrin derivatives, texapyrins, thiopurine derivatives, bacteriochlorin analogs, phthalocyanines and 5-aminolevulinic acid. These compounds are activated at a wavelength >600nm, show greater efficiency in the formation of singlet oxygen and seem to be more effective (Kwiatkowski et al., 2018). Third-generation photosensitizers show greater selectivity for tumor cells and have minimal accumulation in normal tissue. This group includes combinations of photosensitizers of the second generation with molecules targeting the tumor cell receptor, a combination with LDL lipoprotein, a monoclonal antibody targeting a specific antigen or tumor surface markers (such as growth factor receptors, transferrins or some hormones) (Josefsen et al., 2008).

#### 3.4.1 Other photosensitizers in PDT

Turubanova et al. used a standard mixture of PS (di-, tri- and tetrasubstituted fractions of aluminum phthalocyanine) and PD (bis-N-methylglucoamine chloride e6) at a concentra-tion of 10 μg/ml as photosensitizers. Their behavior under the influence of light with wavelengths between 320 and 850 nm for PS and 300–700 nm for PD was studied, re-spectively. *In vitro* studies on the mouse glioblastoma line GL261 have shown that both PS-PDT and PD-PDT are strong inducers of cancer cell death at a light dose of 20 J/cm2 (λex 615–635 nm) (Turubanova et al., 2019). Similar results to 5-ALA are also achieved using 10–20 times lower concentrations of lipophilic 5-aminolevulinic acid esters, such as benzyl-ALA and hex-yl-ALA. This property may result from increased penetration through the cell membrane (Hirschberg et al., 2002). Another potential photosensitizer in PDT of brain glioma may be TiO(2)/PEG, i.e., a combination of polyethylene glycol with titanium oxide, the therapeutic effect of which was confirmed on the spheroids of the rat C6 glioma line (Yamaguchi et al., 2010). Verteporphyrin (VP) may also be of importance as a potential drug in the treatment of glioblastoma of the brain, which, at marginal concentrations and when treated with a 689 nm laser beam, turns out to be a good photosensitizer. This was proved by the *in vitro* studies of Jeising et al., in which the effect of VP on the LN229 and HSR-GBM1 glioblastoma cell lines was examined (Jeising et al., 2022). Among others, sodium sinoporphyrin and me-so-tetra[3-(N,N-diethyl)aminomethyl-4-methoxy]phenylchlorone (TMPC) may be of importance as photosensitizers in PDT of brain glioma (An et al., 2020; Wang et al., 2015). Many other potential photosensitizers may prove to be good alternatives to 5-ALA and hematoporphyrin in the future. More research is needed to prove or disprove their effectiveness.

The photosensitizer is usually administered intravenously and the substance accumulates in the target cancer cells (Eljamel, 2008; Eljamel, 2010; Quirk et al., 2015; Mahmoudi et al., 2019; Bartusik-Aebisher et al., 2022). In the case of GBM, there are 2 methods of irradiating the tumor with a laser of a specific wavelength: intracavitary PDT and interstitial PDT (iPDT) (Dupont et al., 2019; Vermandel et al., 2021). Initially, argon-dye and xenon lasers were widely used, then around 2000, diode lasers were introduced, which are still used today. An alternative is light-emitting diodes (LEDs). They have recently been shown to be as effective and less expensive than their traditional counterparts. Optical fiber devices with cylindrical ends of dispersing fibers are also used (Schmidt et al., 2004). In the case of intracavitary PDT, an expandable irradiating balloon filled with a diluted liquid photodistributor is used and introduced into the operating cavity after prior surgical resection of the primary tumor. In turn, iPDT is a minimally invasive method used in the case of inoperable or recurrent GBM tumors. It consists of stereotactic placement of the previously mentioned scattering fibers directly in the brain tissue (Powers and Brown, 1986; Zhan et al., 2018). After irradiation, the photosensitizer is activated - energy is transferred from PS to molecular oxygen to produce ROS. These reactions occur directly at the irradiated site, sparing healthy tissues. The correlation between the width of the emission band of the light source and the width of the absorption band of the dye is important (Dolmans et al., 2003). The photosensitizer can be administered in various ways, intravenously or topically on the skin, which affects its biodistribution. Upon absorption of light (photons), the sensitizer is converted from its ground state (singlet state) to a relatively long-lasting excited state (triplet state) via a short-lived excited singlet state. The excited triplet form can undergo two types of reactions. Type I is a direct reaction of an excited triplet with a substrate: a cell membrane or a molecule forming radicals such as hydroxy radical that are able to damage cellular structured. In Type II reactions, thetriplet transfers its energy directly to the oxygen to form highly reactive singlet oxygen. Both types of reactions can occur simultaneously, however, for tissue-based PS it is assumed that mechanism II is dominant and determines the effectiveness of therapy. The ROS generated is influenced by substrate and oxygen concentration, pH of the environment and dye quantum yield (Gomer and Razum, 1984; Henderson and Dougherty, 1992; Pass, 1993; Luksiene, 2003; Vrouenraets et al., 2003; Allison et al., 2006). Photodynamic therapy uses different wavelengths of visible light depending on the photosensitizer and its absorption range, as well as the desired depth of penetration of the light into the tissue. The general wavelength range is from about 405 to 900 nm (Quirk et al., 2015). When it comes to photosensitizers, they must meet several conditions: they must be systemically non-toxic, concentrate in the cancerous tissue, absorb light of the appropriate wavelength and not cause damage to the adjacent healthy tissues.

**FIGURE 4:**
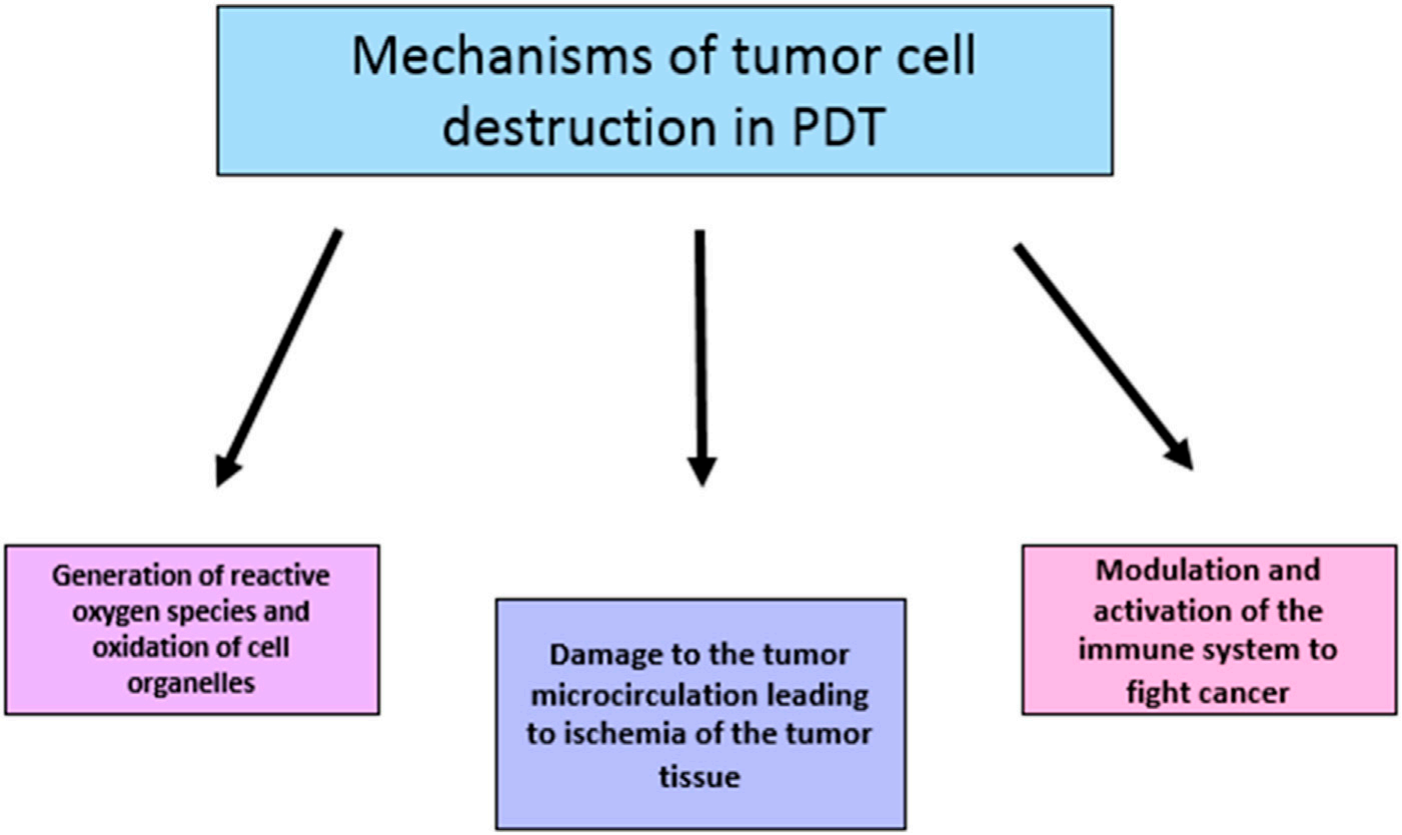
A diagram showing the mechanisms of destroying cancer cells by PDT.

So far, the following PS have been used for this purpose: hematoporphyrin and its derivative HpD, photophrin, boron porphyrin, talaporfin sodium, metatetrahydroxyphenylchlorin (mTHPC) and metabolic precursors of protoporphyrin, such as 5-aminolevulinic acid. These substances can be divided into 3 generations - the first includes natural porphyrins, hematoporphyrins and their derivatives, the second includes chlorins - sodium talaporfin and temoporfin, and the third generation consists of nanoparticles carrying a photosensitizer conjugated with molecules facilitating tumor targeting (Josefsen and Boyle, 2008; Kou et al., 2017; Stallivieri et al., 2018). Photodynamic therapy can destroy cancer cells in three ways. Due to the generation of reactive oxygen species, such as singlet molecular oxygen, hydroxyl radicals and/or superoxide anions, tumor cells are directly destroyed by oxidation of the constituent cell organelles. Cell death can occur through necrosis, apoptosis, autophagy, necroptosis and paraptosis (Kessel, 2019). However, the effectiveness of this method depends on several factors: even distribution of the photosensitizer within the tumor, the distance between the tumor and blood vessels in the case of intravenous administration of the photosensitizer, and the availability of oxygen in the tissue (Tromberg et al., 1990; Korbelik and Krosl, 1994; Messmann et al., 1995; Pogue and Hasan, 1997). Another anticancer mechanism of PDT is the destruction of the tumor microcirculation. Documented studies on PDT with the use of such photosensitizers as porfimer sodium, pyrophorbide derivatives and benzoporphyrin derivatives indicated narrowing of tumor microcirculation vessels, as well as thrombus formation leading to reduced blood supply to the diseased tissue, and thus, its death. On the other hand, the use of PDT contributed to the increased expression of angiogenic factors such as vascular endothelial growth factor (VEGF) and cyclooxygenase (COX)-2, but this fact is not often mentioned in the literature and seems to be less important than the positive effect of PDT on the destruction of tumor vascularization (Henderson and Fingar, 1989; Chen et al., 1996; Fingar et al., 1997; Fingar et al., 1999; Ferrario et al., 2000; Busch et al., 2002; Dolmans et al., 2002; Ferrario et al., 2002). The third mode of action of PDT is participation in the activation of the immune system, which leads to the influx of immune cells into the tumor tissue: leukocytes, lymphocytes and macrophages, and triggers an inflammatory reaction. The process also involves vasoactive substances, components of complement and coagulation cascades, acute phase proteins, proteinases, peroxidases, ROS, leukocyte chemo-attractants, cytokines, growth factors, as well as inflammatory interleukins IL-1 and IL-6. The accumulation of neutrophils has also been found to limit the rate of tumor development (Shumaker and Hetzel, 1987; de Vree et al., 1996; Gollnick et al., 1997). In addition, PTD contributes to the development of an immune response directed against cancer cells, by, e.g., release of tumor antigens from damaged cells. This leads to the stimulation of antigen-presenting cells, such as dendritic cells, which in turn enables the next stages of “setting” the body to fight the diseased cells (Etminan et al., 2011; Li et al., 2011). This is related to the recently discovered use of PDT in the temporary opening of the blood-brain barrier (OBBB). This creates new perspectives for the delivery of drugs to the CNS, which is currently difficult due to the BBB. According to new reports, OBBB enables the activation of lymphatic drainage of brain tissues through the lymphatic vessels of the meninges. Cells and macromolecules, including cancer antigen-presenting cells from PDT-damaged GBM cells, are removed via this route from the CNS to the deep cervical lymph nodes. In the lymph nodes, antigens are presented and CD4+/CD8+ lymphocytes are activated, which migrate back to the vicinity of the tumor and stimulate the destruction of its mass (Semyachkina-Glushkovskaya et al., 2018; Hu et al., 2020; Kanamori and Kipnis, 2020; Semyachkina-Glushkovskaya et al., 2020). Currently, due to the immunomodulatory functions of PDT, research is being conducted on the issue of using GBM cells changed during therapy as a vaccine for this type of glioblastoma (Hirschberg et al., 2018; Madsen et al., 2018). Further research is needed to visualize the distribution of photosensitizers in the body, as well as to improve the methods of measuring the amount of oxygen produced necessary to destroy cancer cells (Mahmoudi et al., 2019). Limitations of PDT include the fact that the remaining, undegraded photosensitizer must be removed from the body, and the possibility of photosensitivity of the skin after its administration. In addition, destruction of hypoxic tumors may be hindered by the low availability of oxygen for the necessary reactions. These problems are likely to be solved in the near future (Eljamel, 2010; Quirk et al., 2015; Mahmoudi et al., 2019; Bartusik-Aebisher et al., 2022). Figure 4 shows mechanisms of destroying cancer cells by PDT.

### 3.5 Standard methods in the treatment of a brain tumor

Treatment methods for glioblastoma aim to remove the tumor completely or at least reduce its size in order to alleviate the symptoms and prolong the patient’s life with as much comfort as possible. The choice of method depends on many factors, including the type, size and location of the tumor, as well as age, medical history, general health, and the intensity and nature of the symptoms observed in the patient. Standard therapy is based on three methods: surgery, radio-(RT) and chemotherapy (Shah and Kochar, 2018). Surgical resection is usually used in the early stages of cancer, especially in the case of benign tumors, because then it can ensure a complete cure. The main limitation of this method is that many tumors are located in places where access is limited, and interference could result in damage to important brain structures with serious health consequences for the patient. Complications may also occur after a properly performed procedure. Moreover, often the patient’s general condition does not allow for surgical intervention with the use of general anesthesia (Shah and Kochar, 2018). Another method is radiotherapy, which is based on the destruction of cancer cells using ionizing radiation - photon, electron or proton. Radiation causes ionization of molecules in the irradiated tissue, which results in DNA damage and cell death. Radiation energy is absorbed directly by cellular structures: DNA, organelles and cell membrane or indirectly by induction of highly reactive free radicals in the cytosol. This process is more effective in an environment rich in molecular oxygen. Due to the method of delivering RT radiation, it can be divided into brachytherapy, where the source of rays is located in the immediate vicinity of the tumor or inside it, which allows for precise delivery of a large dose to the desired structure, and teleradiotherapy, where the irradiation of tissues takes place from a certain distance (with using a linear accelerator as a source of photons). A more precise method, derived from teleradiotherapy, are stereotactic techniques, also referred to as stereotactic radiosurgery using, for example, a gamma knife [GK]. Radiotherapy is used to treat most types of brain tumors, but with varying success - for example, glioblastomas tend to progress despite high doses of radiation. Radiotherapy is also associated with many annoying side effects, such as fatigue, headaches, nausea and hair loss (Laperriere and Bernstein, 1994; Johnson et al., 2008; Minniti et al., 2012). Another standard method of treatment is chemotherapy, based on the administration of cytostatic drugs that inhibit cell division. Its main limitations include the systemic toxic effect of the compounds used and the disruption of the differentiation of healthy cells, e.g., in the bone marrow, which results in a reduction in the number of immune cells and impairment of the body’s immune functions. Standard first-line chemotherapy involves the use of temozolomide (75 mg/m^2^ daily) during radiotherapy, followed by 6 consecutive cycles of this drug (150–200 mg/m^2^ on days 1–5 every 28 days). The most common side effects are nausea, thrombocytopenia and neutropenia. Usually, after the first course of treatment, the disease recurs within 6 months. In the second line, alkylating chemotherapy is usually used: lomustine, carmustine, and a second attempt at temozolomide is made. The effectiveness of chemotherapeutics is often reduced by the need to cross the blood-brain barrier and limited accumulation in tumor cells (Tan et al., 2020; Saito, 2021).

### 3.6 Targeted methods in the treatment of a brain tumor

Targeted therapy aims to increase the precision of cancer therapy by increasing the toxicity of drugs used for diseased cells while sparing healthy tissues. Such selective action can be achieved by using receptors on cancer cells and absent or present in a reduced amount on healthy cells. It is, among others in the case of LDL receptors, which are used to deliver photosensitizers to cancer cells (Gutman et al., 2000). Glioblastoma cells also differ from normal cells by the presence or overexpression of interleukin-13 (IL13) receptors, fibroblast growth factor (FGF) receptor, Neu/ErbB2 receptor, tumor-specific antigens including MAGE, and tumor-associated extracellular matrix proteins, such as chondroitin sulfate proteoglycan. These and other receptors can become a target for appropriately modified antibodies, which enables new directions of therapy (Gutman et al., 2000). Methods that target enzymes or growth factors crucial for tumor division and growth, such as tyrosine kinase (TKI) or vascular endothelial growth factor (VEGF), are also being investigated. Monoclonal antibodies such as Bevacizumab, which binds to vascular endothelial growth factor (VEGF) and thus slows down tumor angiogenesis, are used here. It is often used in combination with chemotherapy (Diaz et al., 2017). It is well known that in cancer cells there are mutations in the p53 protein gene as well as in the genetic material of the MDM2 and MDM4 proteins that regulate it, so that p53 does not perform its functions in controlling the cell cycle and preventing excessive cell proliferation. For this reason, inhibitors of defective proteins are being developed, such as the MDM2-AMG 232 inhibitor, which can be used to slow down tumor progression (Yang et al., 2022). Glioblastoma immunotherapy, on the other hand, focuses on stimulating the immune system in order to “tune” it to fight the diseased cells, which is related to the work on cancer vaccines. In addition, research is being conducted on the tumor microenvironment (TME), consisting of immune cells (mostly macrophages), fibroblasts, endothelial cells, extracellular matrix (ECM), vascular system and chemokines. TAMs is an abbreviation for tumor-associated macrophages and microglial cells that are recruited into the tumor environment to produce factors that promote tumor growth, including by promoting angiogenesis. These macrophages have receptors for the colony-forming factor CSF-1R on their surface, which can be blocked using specific inhibitors, e.g., BLZ945 and PLX3397. Another method of affecting immune cells is tumor antigen vaccines designed to stimulate dendritic cells (e.g., DCVax-L) and other APCs. Antigens can be obtained, for example, from heat shock proteins or purified peptides derived from tumor cells, as in the case of the NCT01814813 and NCT03018288 vaccines (Miyauchi and Tsirka, 2018). After contact with a foreign antigen, dendritic cells present it to T lymphocytes, which mobilizes them to destroy cancer cells. An example of a DC vaccine is ICT-107, which sensitizes APCs to tumor antigens such as HER2, TRP-2, gp100, MAGE-1, IL13Rα2 and AIM-2. DC vaccination may contribute to the prolongation of patients’ lives and prolongation of the disease progression-free period (Yang et al., 2015; Hoang-Minh and Mitchell, 2018; Miyauchi and Tsirka, 2018). An important discovery was also the development of immune checkpoint inhibitors.

So-called checkpoints, i.e., PD-1 and CTLA-4 molecules, are found on the surface of T and B lymphocytes. In the case of chronic inflammation, as well as in the course of cancer, these receptors are upregulated, which by connecting with their ligands (PD-L1) present on cancer cells, macrophages and APCs within the tumor cause the inhibition of proliferation and apoptosis of T lymphocytes (except for Tregs). All this contributes to a decrease in the population of Th and Tc lymphocytes, an increase in the number of Tregs, and thus immunosuppression favoring the development of cancer. For this reason, the discovery of checkpoint inhibitors: anti-CTLA-4 (e.g., ipilimumab) and anti-PD-1/PD-L1 (e.g., nivolumab, pembrolizumab) ICIs seems to be a promising method in the treatment of glioblastoma (Prins et al., 2011; Preus et al., 2015; Berghoff et al., 2016; Butowski et al., 2016; Quail and Joyce, 2017). Another direction of immunotherapy, which is gaining more and more importance, are CAR-T, i.e., modified T lymphocytes, taken from a patient or a healthy donor, to which the CAR receptor is attached, which can recognize cancer antigens, bind to them and activate the T lymphocyte, thus cancer cells are destroyed in different ways. The chimeric CAR itself consists of an extracellular antigen-sensing domain and an intracellular signaling-lymphocyte-activating domain linked by a transmembrane linker. In the case of brain tumors, the following are considered as targets for modified lymphocytes: EGRFvIII - mutated epidermal growth factor receptor (EGFR), which is the most common variant of this receptor in cancer cells, IL13Rα2 - receptor for IL-13, which is expressed in more than 75% of GBM and heralds a high aggressiveness of the tumor and poor prognosis, HER-2, i.e., a receptor for human epidermal growth factor, which is overexpressed in many types of cancer and in about 80% of GBM, as well as such cancer antigens as B7-H3, CD147 or GD2. The limitation of CAR-T therapy are difficulties in the delivery and accumulation of lymphocytes in the tumor tissue due to the need to penetrate the blood-brain barrier. The solution may be to place the modified cells directly in the tumor mass or in the cavity after its resection. CAR-T is often combined with other methods of treatment, such as radio- and chemotherapy, and other forms of immunotherapy in order to achieve a better therapeutic effect (Ch et al., 2020; Lindo et al., 2021; Marei et al., 2021). An interesting method is also gene therapy, which uses various vectors, including viruses, to introduce changes in the genetic material of cancer cells and thus to destroy them. The introduction of modified genes contributes to the damage of cancer cells in various ways. It may be cytotoxic, directly causing cell death by blocking DNA synthesis, as well as restoring mutated tumor suppressor genes to normal function, thereby controlling the cell cycle and preventing excessive cell proliferation, as well as blocking tumor angiogenesis. Modified viruses can also lead to the destruction of cancer cells themselves. Viruses that are being studied for adaptation to therapy include: Delta-24-RGD adenovirus (DNX-2401), measles virus, herpes simplex virus and polio virus PVS-RIPO (NCT01491893), as well as genetically modified HSV, reoviruses and Newcastle disease virus. Glioblastoma cells are characterized by an increased expression of the CD155 receptor, which is recognized by poliovirus. When combined, PVS-RIPO leads to immunogenic cell death with the release of tumor antigens, which are taken up by dendritic cells and presented to T lymphocytes, thereby stimulating the immune system to fight the tumor.

**FIGURE 5:**
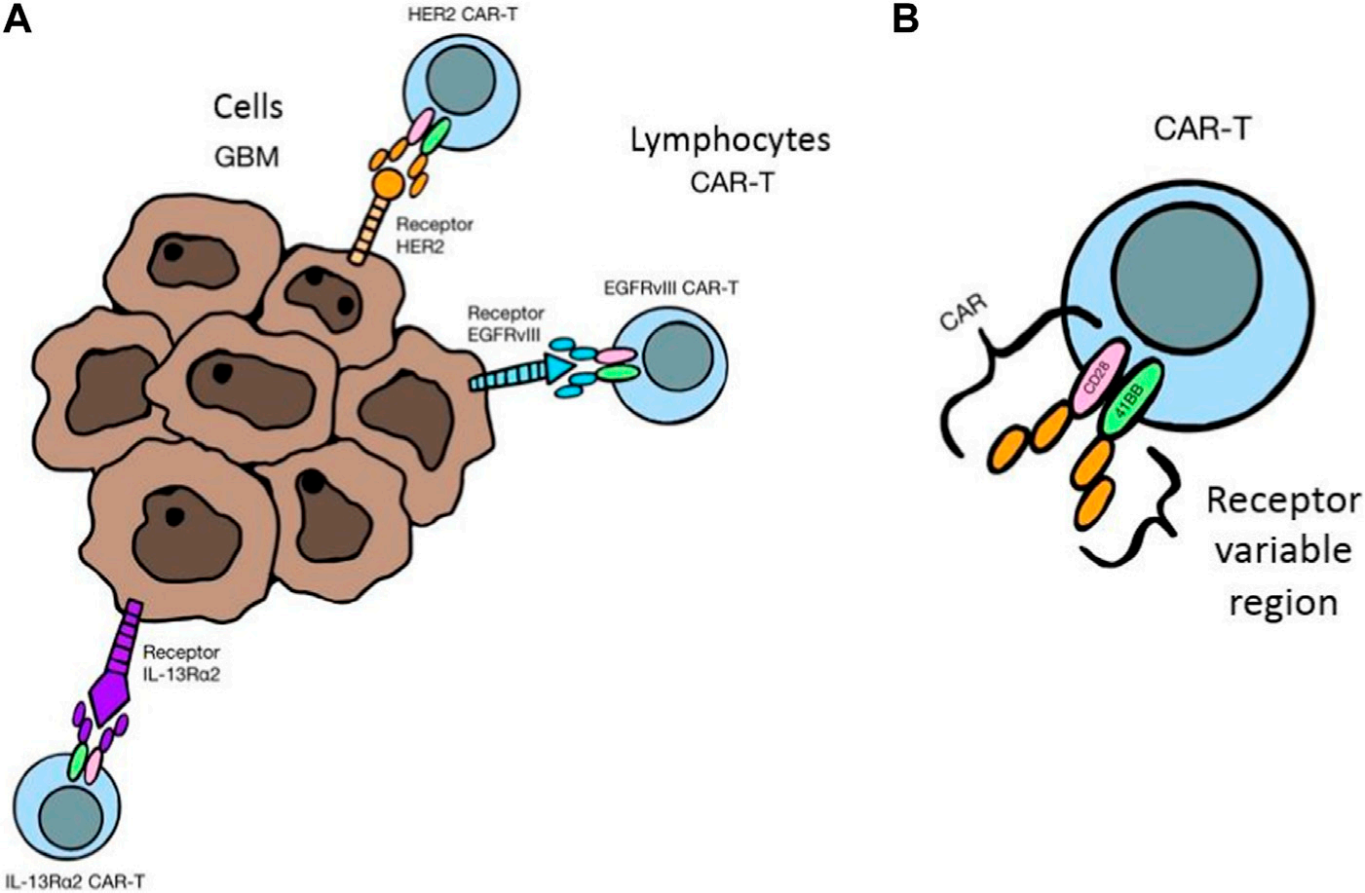
**(A)** Mechanism of selective attachment of CAR-T lymphocytes to tumor cell receptors, 3 **(B)**. Structure of a modified T lymphocyte with the CAR receptor.

The destruction of tumor cells is also carried out by implanting the genetic material of the virus into their genome, e.g., thymidine kinase gene. Then, antiviral drugs are administered, e.g., ganciclovir, which destroys the cells that produce viral proteins. In turn, the use of modified adenoviruses expressing IL-12 may lead to the transformation of TAMs into anticancer phenotypes (Dunn-Pirio and Vlahovic, 2017; Maxwell et al., 2017). Figure 5A shows mechanism of selective attachment of CAR-T lymphocytes to tumor cell receptors and Figure 5B structure of a modified T lymphocyte with the CAR receptor.

#### 3.6.1 Nanoparticles supporting PDT

**FIGURE 6:**
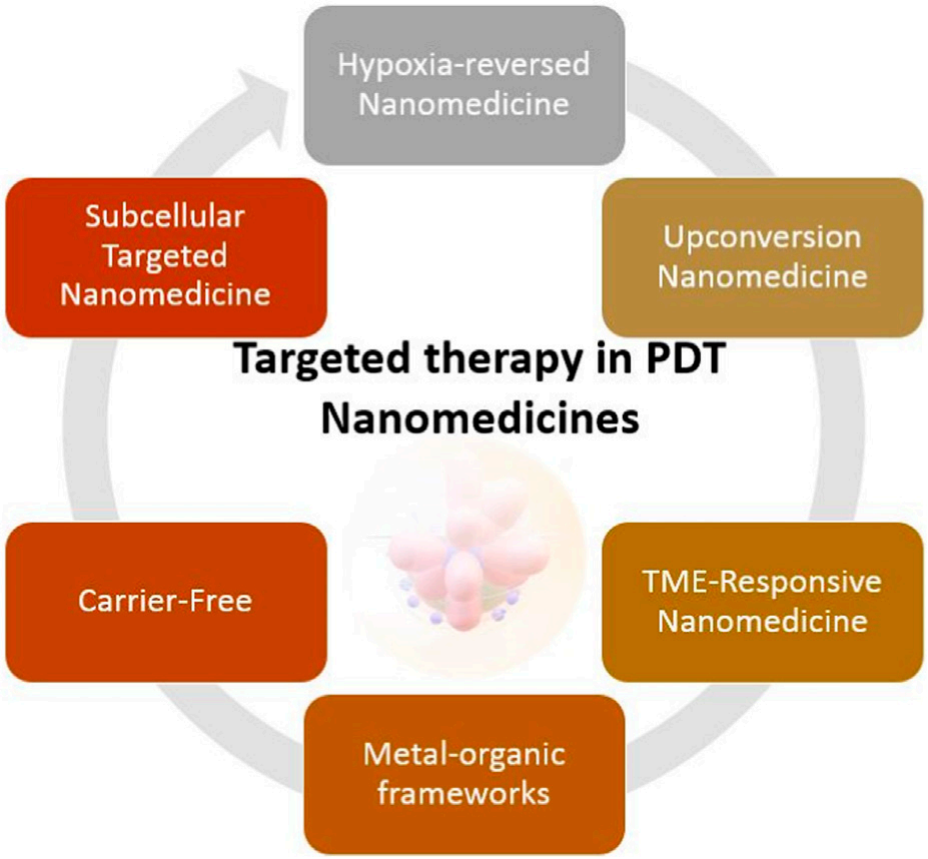
Examples of nanostructures used in PDT.

More and more often, in combination with PDT, nanocomposites and nanoparticles are used to support the effectiveness of therapy (Jin et al., 2021). Research shows that the use of various elements of nanomedicine improves treatment results. Below are some examples of nanocomposites supporting PDT. One example is nanoparticles that reverse tumor hypoxia (Wang et al., 2019). This group of nanoparticles includes quinones and nitroimidazoles. Nanoparticles consisting of quinones are selectively activated in a site with limited oxygen saturation, mainly in the environment of tumor cells. In turn, nitroimidazole is usually one of the elements developed in PDT. May be part of a complex in combination with ROS (Qian et al., 2016). Another example is upconverting nanoparticles (UCNPs). According to Zhang et al., the upconversion of Au-doped nanoparticles has a relatively high light intensity. In particular, Au-doped UCNPs are non-toxic to tissues, making them extremely effective (Zhang et al., 2022). In turn, the Tumor Microenvironment (TME)-Responsive nanoparticles group includes such platforms as: nanogels, hybrid micelles, nanocoatings, and miceplexes. According to Qin et al., nanogels are an effective and safe method of drug delivery in therapy (Qin et al., 2020). Just like hybrid micelles or miceplexes. Chemo-photodynamic therapy and the use of organometallic structures in combination with photosensitizers are gaining in popularity. Designed and applied by Zheng et al., complexes of nanoparticles with organometallic substrates had high anticancer activity and satisfactory biocompatibility (Zeng et al., 2021). Another example is carrier-free nanoplatforms. According to Ji et al., the nanoplatform designed in this way allows the drug to be applied for the application of the photosensitizer without the occurrence of potential side effects. Another example is the study carried out by Wang et al., who constructed a carrier-free nanoplatform in combination with ICG and αPD-L1, which can self-assemble into nanoparticles (Wang et al., 2019). The last mentioned group of nanoparticles are subcellular targeted nanoparticles in PDT. The principle of operation of these consists in targeted targeting of cellular structures, i.e., mitochondria, lysosomes, cell nuclei or the plasma membrane. According to Ji et al., central delivery of photosensitizers to, e.g., mitochondria using a nanocomposite may be a potential strategy to enhance cancer immunotherapy (Ji et al., 2022). Figure 6 shows examples of nanostructures used in PDT.

There is still a need for studies evaluating the correlation of PDT with various nanocomposites or nanostructures in combination with applied photosensitizers. Advanced developed structures in the field of nanomedicine can be a promising and, above all, a common therapeutic tool.

#### 3.6.2 The advantages and the disadvantages of PDT compared to the other therapies

Surgical resection is the most common tool in cancer treatment (Juvekar et al., 2023). Its highest effectiveness occurs when applied in the early stages. Despite its commonness, it has several challenges and problems that PDT can solve. The first is the location of the tumor in the patient’s body. Tumors located deep or in hard-to-reach places are a challenge for surgeons. In addition, organs made of connective tissue are localized and constitute an important structure, such as bronchi or the brain. In such cases, the doctor does not decide to perform resection. In such cases, minimally invasive methods such as PDT are necessary. PDT as a therapeutic method damages cancer cells, leaving the collagen structure intact (Overchuk et al., 2023). Therefore, in cases where resection is impossible, PDT is applied. Another disadvantage of surgical resection are the side effects and complications after the procedure, i.e., nausea, problems with the healing wound, pain (Gan, 2017). In turn, PDT has fewer side effects and the wounds after the procedure are very small or do not occur at all. Another aspect of resection is that when removing cancerous tissues, the doctor also removes a several millimeter margin of healthy tissue. The solution is PDT, which works selectively. The applied photosensitizer selectively accumulates in cancer cells, and not in the surrounding healthy cells without signs of disease. Therefore, PDT is a cancer-selective method (Hong et al., 2016). The last aspect is the whole resection procedure. It requires prior preparation. In contrast, PDT can be used on an outpatient basis. Compared to radiotherapy, PDT can be used many times, so it is reproducible. In addition, like surgery, radiotherapy is associated with side effects, which makes PDT an alternative in this aspect. The most crucial difference between PDT and radiotherapy is that it takes work to assess dosimetry at the time of treatment in PDT accurately. Furthermore PDT compared to radiotherapy uses nonionizing radiation (Zhu and Finlay, 2008). It can be used for a long time, in stages or sequentially. Therefore, it is necessary to establish a method to evaluate the therapeutic effect on tumor cells and side effects on normal brain cells with high reliability. Compared to chemotherapy, PDT does not work systemically, but locally. Thanks to this, the patient is not exposed to adverse side effects. In turn, immunotherapy is a very expensive and more expensive method than other forms. In turn, PDT generates lower costs, which makes it widely applicable. In addition, immunotherapy may correlate with other applied therapies, which reduces the effectiveness of treatment. According to Calixto et al., one of the main disadvantages of PDT is the hypersensitivity of patients to light after treatment. This aspect does not appear with other methods. In addition, at the moment there is no clearly defined effective dose of the photosensitizer and the time of exposure to light. The effectiveness of the therapy depends on the most precise delivery of the light source. In PDT, it is also necessary to properly oxygenate the treated tissue, which is not the case in other therapies. Additionally, PDT is not used to treat metastatic cancer (Calixto et al., 2016).

## 4 Summary

Malignant tumors of the brain and central nervous system (CNS) are a serious threat to health and cause the death of people. Current standard treatments for brain tumors have some limitations. New therapeutic methods are constantly being sought. One such solution is PDT. The main principle of operation of PDT is based on the effect of combining photosensitizer molecules with cancer cells and their activation by excitation with laser light of the appropriate wavelength. The main advantage of this method is its high precision—cancer tissue or tumor vasculature containing PS can be selectively irradiated sparing healthy tissue. Photodynamic therapy was first approved in 1993 using the photosensitizer Photofrin for the treatment of bladder cancer. Since then, many papers have been published confirming the advantages of PDT in the treatment of gliomas and showing its advantage over standard surgical therapy. Currently, in the PDT treatment of brain tumors, the selection of the photosensitizer and, consequently, the selection of the light source still remains a challenge. Therefore, the solution may be targeted therapies or the use of nanocomposites.

## 5 Conclusion

Brain tumors, including glioblastoma multiforme, are currently the cause of suffering and death of tens of thousands of people worldwide. Despite the progress of medicine, the average patient survival from the moment of diagnosis of GBM with the use of standard treatment methods, such as surgical resection, radio- and chemotherapy, is less than 4 years. The development of new therapeutic methods targeting brain tumors may extend life and provide greater comfort to patients. Particular attention should be paid to photodynamic therapy, the effectiveness of which in the treatment of GBM, resulting in prolongation of progression-free survival, has been confirmed in many scientific studies (although still leaving the fact of the effectiveness of photosensitizers to be improved). Another important aspect is the ongoing development of immunological therapies for brain tumors, which have already been used in the treatment of other cancers with positive results. Cancer vaccines, monoclonal antibodies, gene therapies or modified CAR-T lymphocytes form a dynamically progressing branch of medicine, which is of interest to many scientists and may revolutionize the future of brain tumor treatment.
